# Urinary tract infection associated with bacteremia caused by vancomycin‐resistant enterococcus following continent urinary diversion

**DOI:** 10.1002/ccr3.8214

**Published:** 2023-12-28

**Authors:** Kai Morishita, Soichiro Kashiwabara, Yuki Matsumoto, Atsuhiro Mizushima, Kaori Hara, Toshihiko Agatsuma, Yuji Mimura, Hideyoshi Matsumura, Hiroya Mizusawa

**Affiliations:** ^1^ Department of Urology National Hospital Organization, Shinshu Ueda Medical Center Nagano Japan; ^2^ Department of Gynecology National Hospital Organization, Shinshu Ueda Medical Center Nagano Japan; ^3^ Department of Pharmacy National Hospital Organization, Shinshu Ueda Medical Center Nagano Japan; ^4^ Department of Infection Control National Hospital Organization, Shinshu Ueda Medical Center Nagano Japan

**Keywords:** continent reservoir, in‐hospital outbreak, multidrug‐resistant bacterium, postoperative complication, pyelonephritis

## Abstract

**Key Clinical Message:**

Even in a country where vancomycin–resistant enterococcus is rare, multidrug‐resistant organism precautions are necessary when admitting patients with a history of medical exposure in other countries. On admission, screening is necessary and if infection is confirmed, a multidisciplinary approach involving different specialists is required.

**Abstract:**

The patient was a 49‐year‐old Japanese female living in the United States. Total pelvic exenteration for cervical carcinoma, Miami pouch formation, and ileostomy had been performed in the United States. She returned to Japan to undergo postoperative adjuvant chemotherapy. Fever and abdominal pain occurred 42 days after surgery. She consulted the fever outpatient clinic, and a diagnosis of urinary retention‐associated acute renal failure and pyelonephritis was made. We detected vancomycin‐resistant enterococcus on urine/blood culture 5 days after admission. Infection control measures were implemented, and the ward was closed for 3 days. We administered linezolid, which was effective for pyelonephritis and bacteremia.

## Graphical abstract

Miami pouch in the right lower abdomen is dilated because of incomplete intermittent self‐catheterization. It caused pyelonephritis and bacteremia by vancomycin‐resistant enterococcus.

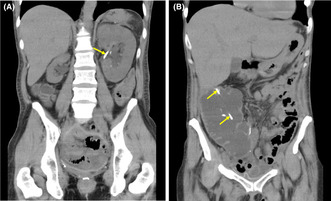

## 1 INTRODUCTION

The proportion of enterococcal infections that are resistant to vancomycin varies by country. Vancomycin‐resistant enterococcus (VRE) is rare in Japan.^1^, ^2^, ^3^ In this study, we report a patient with urinary tract infection associated with VRE following continent urinary diversion surgery abroad. As there was a risk of in‐hospital outbreak, infection control was necessary.

## 2 CASE PRESENTATION

The patient was a 49‐year‐old Japanese female living in the United States. She complained of fever and abdominal pain. Her medical history was not contributory. She had no risks for infection except for malignant disease and postoperative condition.

**FIGURE 1:**
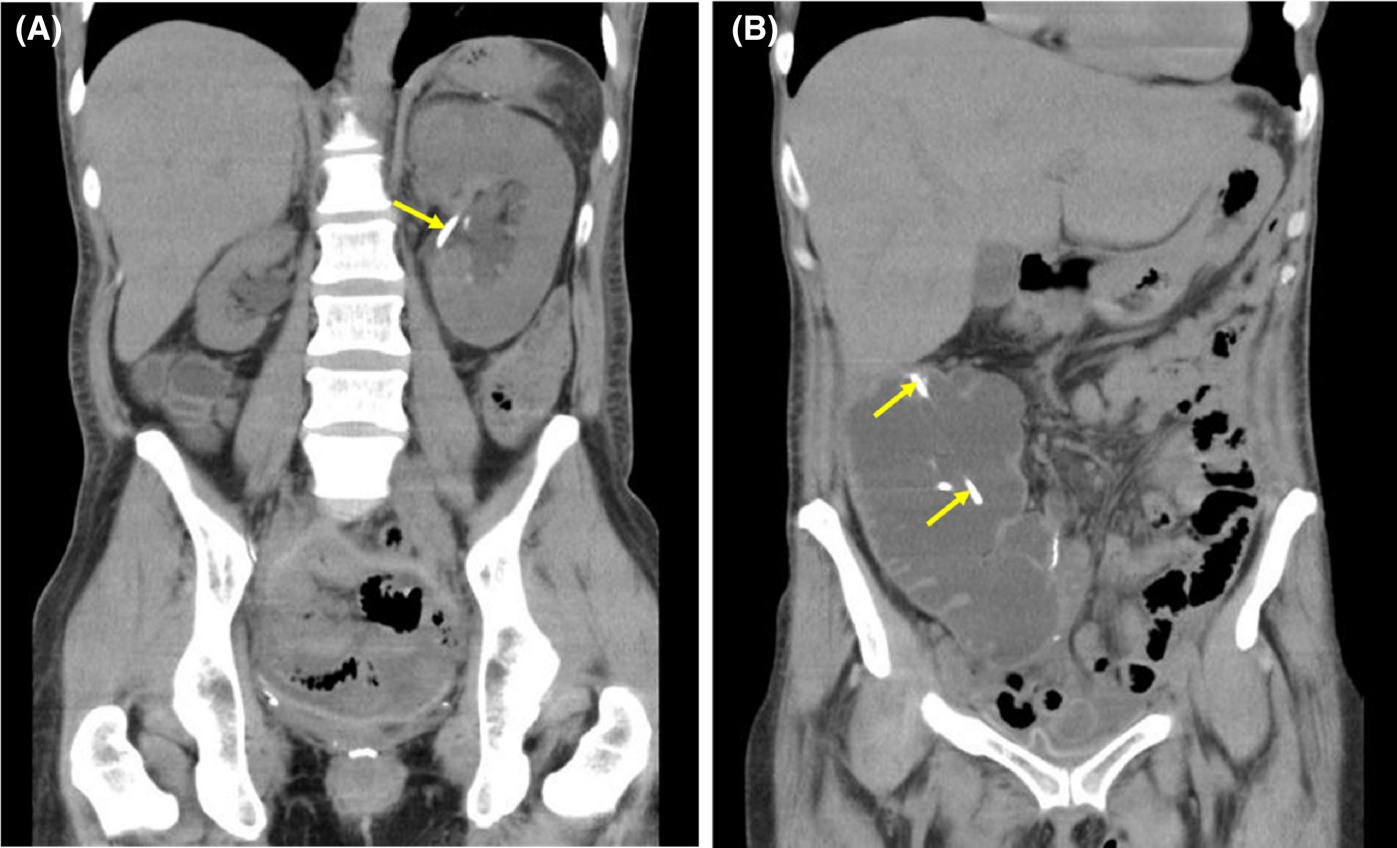
Plain computed tomography on the initial consultation. Bilateral mild hydronephrosis, right renal atrophy (A), and marked dilation of a urinary pouch (B) are observed. A ureteral stent (arrows) had been placed.

Total pelvic exenteration for cervical carcinoma, Miami pouch formation, and ileostomy (SCCpT3bN1aM1) had been performed in the United States. She returned to Japan to undergo postoperative adjuvant chemotherapy. Fever and abdominal pain occurred 42 days after surgery, and she consulted the fever outpatient clinic of our hospital before a referral reservation. Abdominal computed tomography (CT) revealed enlargement of the Miami pouch, bilateral mild hydronephrosis, and right renal atrophy. Furthermore, we observed bilateral stents (Figure 1). Blood biochemistry showed severe renal failure and severe bacterial infection (Table 1). A diagnosis of urinary retention‐associated renal failure and pyelonephritis was made based on topical findings and pyuria/bacteriuria. The patient was urgently admitted.

**TABLE 1:** Changes in hematological data.

		On admission	Day 4 of admission	Day 11	Day 15	Day 22
Albumin	g/dL	3.6	2.8	2.7	2.9	3.0
Aspartate aminotransferase	U/L	18	19	17	18	10
Alanine aminotransferase	U/L	25	28	28	31	14
Blood urea nitrogen	mg/dL	50.3	29.3	13.6	12.8	9.0
Creatinine	mg/dL	4.18	2.86	1.83	1.2	1.18
Sodium	mEq/L	136	139	137	140	141
Potassium	mEq/L	5.5	3.9	3.2	3.8	4.1
Chloride	mEq/L	106	111	101	104	104
C‐reactive protein	mg/dL	29.6	23.9	4.7	1.2	0.4
White blood cell	×10^4^μL	161	109	48	65	43
neutrophil	%	84.9	85.6	66.2	48.2	50.8
eosinophil	%	1.2	0.9	18.5	12.2	4.9
Red blood cell	×10^4^μL	408	360	359	362	379
Platelet count	×10^4^μL	49.1	57.9	9.9	83	63.7

On admission, consciousness was clear. Blood pressure, pulse, body temperature, and oxygen saturation (indoor) were 129/76 mmHg, 112 bpm, 40.0°C, and 93%, respectively. The abdomen was flat. There was a surgical wound in the midline of the abdomen. In the right lower abdomen, a urinary stoma was present, and a stent had been placed in the stoma. Tenderness was noted at the site. Ileostomy had been performed in the left abdomen.

We attempted to insert a catheter into the pouch, but insertion was difficult. An endoscope was inserted beside the urinary stoma stent. The efferent tube (ileum with narrowing) was flexed. This may have led to incomplete self‐catheterization, resulting in urinary retention. There was no stenosis. Endoscopy revealed that intra‐pouch urine was markedly turbid, and two stents were observed. The margin of one stent was placed in the pouch. Two stents were left, and a catheter was inserted into the pouch.

**FIGURE 2:**
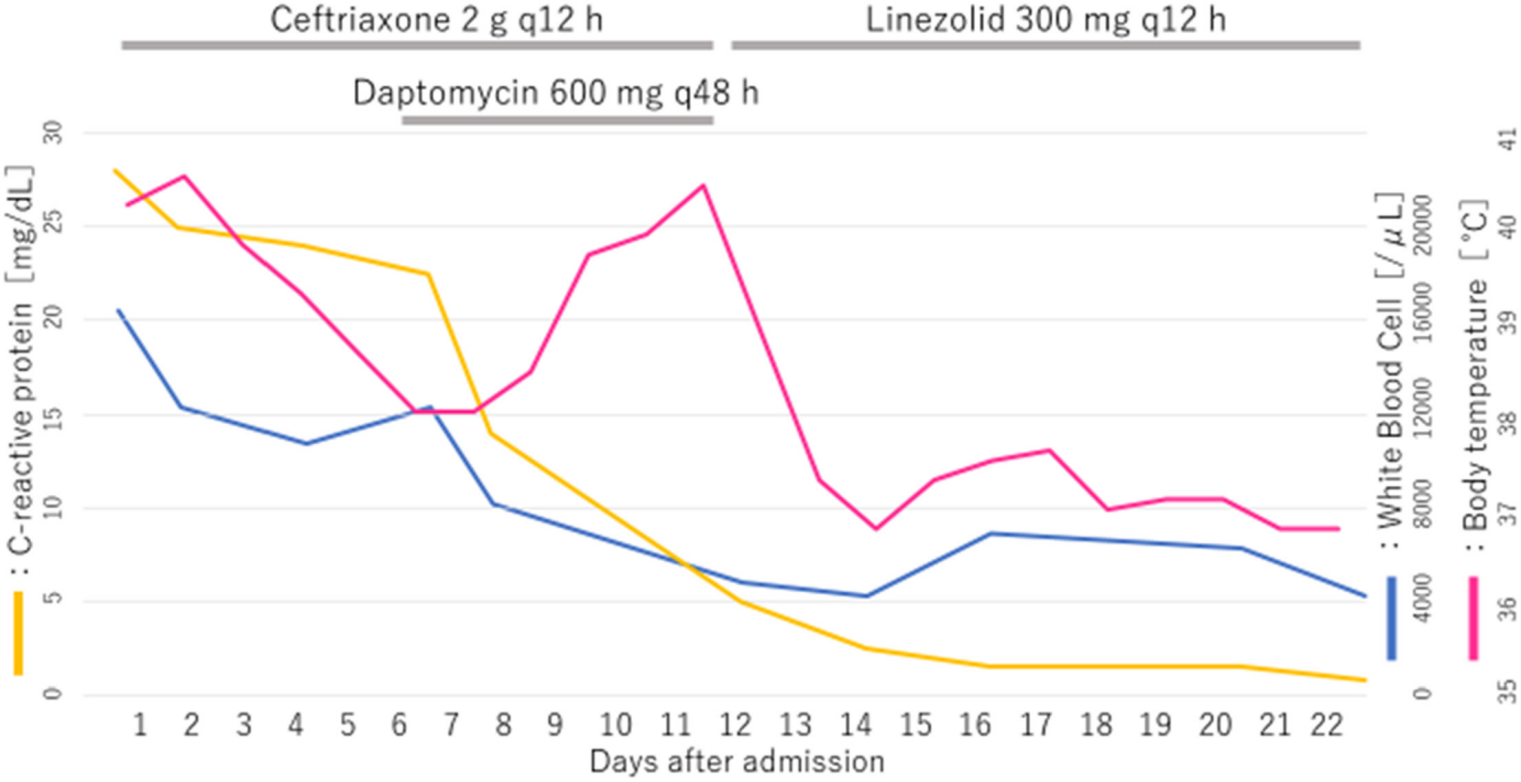
Fever, laboratory data, and course of antimicrobial drug administration.

To treat pyelonephritis, twice‐a‐day ceftriaxone administration at 2 g/session was started. Despite an improvement in drainage from the pouch, fever persisted 4 days after admission (Figure 2), and there was no marked improvement in the hematological data (Table 1).

The detection of VRE on urine/blood culture became clear 5 days after admission. We identified a vancomycin‐resistant *Enterococcus faecium* (>10^5^ CFU/mL). Antimicrobial susceptibility testing demonstrated that it was resistant to penicillin, ampicillin, minocycline, vancomycin, teicoplanin, levofloxacin, and rifampicin, and sensitive to linezolid. Once‐a‐day daptomycin administration at 600 mg (12 mg/kg) at 48‐h intervals was added. On Day 8, bilateral ureteral stents were exchanged. The blood data were gradually improved (Table 1), and the disappearance of pain was achieved on Day 9. On Day 11, erythematous eruption appeared on the trunk and medial side of the upper and lower limbs, accompanied with high fever. According to an assessment by a dermatologist, broad drug exanthema may be involved in high fever. Considering the possibility of an adverse reaction, administration of daptomycin and ceftriaxone was discontinued, and linezolid at 300 mg was administered twice a day. A blood culture test on the same day showed a negative reaction. On Day 14, body temperature was normal. On Day 22, linezolid administration was completed (Figure 2). Contrast‐enhanced abdominal CT confirmed the disappearance of bilateral hydronephrosis; however, we found peritoneal dissemination. On Day 35, the patient was discharged (Table 2).

**TABLE 2:** Timeline of main events.

Day ‐42	Total pelvic exentration and Miami pouch formation for cervical cancer in United States
Day 1	First visit to our hospital for high fever and abdominal pain. CTRX was started on admission
Day 5	Detection of VRE on urine/blood culture. DPT was added
Day 9	Disappearance of abdominal pain
Day 11	Development of broad skin eruption. CTRX and DPT were stopped. LZD was started
Day 13	Confirmation of negative VRE on blood culture
Day 14	Body temperature was normal
Day 22	LZD administration was completed
Day 35	Discharge
Day 42	Chemotherapy was started
Mon 21	Chemotherapy was completed. Change to palliative care policy
Mon 24	Death

Abbreviations: CTRX, ceftriaxone; DPT, daptomycin; LZD, linezolid; VRE, vancomycin‐resistant enterococcus.

Ureteral stents were exchanged 3 months after surgery. The upper end of the right stent had deviated into the pouch, and it was removed. The left ureteral stent was exchanged every 2 months. The interval until stent occlusion was short, and the frequency of stent exchange was changed to once a month. Pyelonephritis possibly associated with occlusion occurred twice; however, it was not a VRE infection. The left ureteral stent was removed 12 months after surgery, and an intra‐pouch catheter was placed. In the bilateral kidneys, Grade 2 hydronephrosis was noted. Subsequently, there was no urinary tract infection‐related fever.

For cervical carcinoma, six courses of therapy with paclitaxel and carboplatin, nine courses of therapy with irinotecan, and four courses of therapy with docetaxel were performed. However, the patient's condition gradually progressed, and the patient died 24 months after surgery.

## 3 DISCUSSION

According to the Centers for Disease Control and Prevention in the United States, VRE accounts for approximately 30% of all types of enterococci,^1^ whereas the percentage is reportedly 0.8% according to data aggregated in 2021 by the Japan Nosocomial Infections Surveillance of the Ministry of Health, Labour and Welfare.^2^ Globally, the VRE isolation rate varies widely. The VRE isolation rate is low in the Asian region overall. It is high in Korea, but low in Japan compared to other countries.^3^, ^4^

In 2019, 80 VRE patients were reported throughout Japan. The male‐to‐female ratio was 1:1, and the median age was 79 years. Bacteria were detected in urine, blood, and ascites samples (38%, 31%, and 9% of samples, respectively). Urinary tract infection was observed in 26 patients (33%), and bacteremia in 20 (25%).^3^

Miami pouch is a continent reservoir using a part of the ascending colon and terminal ileum. The ileocecal valve prevents urinary leakage, and the patient drains their urine by intermittent self‐catheterization. Difficulty of catheterization may occur because of stoma stenosis or flexion of efferent tube.^5^ In the present case, flexion of the efferent tube induced difficulty of catheterization, and retention of the pouch caused pyelonephritis, bacteremia, and severe renal failure.

The pathogenicity of VRE is low, but surgical site infection, urinary tract infection, and bacteremia may occur in patients with underlying diseases; thus, caution is needed. A previous study reported that the admission period was 5 days longer than in patients infected with vancomycin‐susceptible enterococcus, and that the mortality rate was 1.8 times higher.^6^

For the treatment of VRE infection, daptomycin is the first‐choice drug.^7^ In our patient, combination therapy with daptomycin and a β‐lactam was started. Dosage of daptomycin was decided based on the guidelines and severe renal insufficiency.^7^ Later, an adverse reaction to daptomycin was suspected; therefore, it was switched to linezolid. This change was clinically effective.

VRE is highly transmissible, and readily causes contact infection; however, its onset is rare, and it may not be readily noticed. Thus, nosocomial outbreaks may occur. This type of bacteria is important for medical facilities with a large number of high‐risk patients from the viewpoint of infection control. When a patient is confirmed to be positive, we should treat it the same as an outbreak. Standard preventive measures alone are not sufficient; therefore, contact prevention strategies should be adopted such as hospitalizing each patient in a private room. If there are several patients, cohorting and fecal examination should be performed. Fecal screening for health care professionals and inpatients must be reviewed by the Department of Infection Control.^8^

Hayakawa et al.^9^ reported that 56.5% of patients with a history of hospitalization abroad had drug‐resistant bacteria, including ESBL and MRSA, and that these bacteria included highly resistant bacteria such as MDRA and VRE. When admitting patients with a history of medical exposure in other countries, caution is needed, and private room isolation/contact infection control must be performed until the presence or absence of carriage is confirmed.^3^

Examination of multidrug‐resistant bacteria was not performed on admission in the present case; however, our patient was hospitalized in a private room from the time of admission, considering the possibility of severe infection and COVID‐19 from the United States. After VRE infection became clear, contact preventive measures were taken for in‐hospital infection control. Subsequently, the inpatient ward was closed (new admission, ward transfers, and hospital transfers were stopped) after conferences on management by the Department of Infection Control and a health center. A fecal culture test was conducted in all inpatients in the ward to confirm VRE‐negative reactions. The ward closure period was 3 days.

We should consider that the risk of nosocomial infection is high in elderly, cancer, or catheter‐inserted patients.^10^ Currently, we operate infection prevention measures according to the scale of our hospital such as infection screening on admission for patients who were hospitalized abroad within the previous few months.

## 4 CONCLUSION

Even in an area where VRE is rare, screening on admission is necessary for patients who have received treatment in other countries. If infection is confirmed, a multidisciplinary approach, including an oncologist, an infectious disease specialist, and a pharmacist, is necessary.

## AUTHOR CONTRIBUTIONS

**Kai Morishita:** Visualization; writing – original draft. **Soichiro Kashiwabara:** Data curation; writing – original draft. **Yuki Matsumoto:** Data curation. **Atsuhiro Mizushima:** Supervision. **Kaori Hara:** Data curation; supervision. **Toshihiko Agatsuma:** Supervision. **Yuji Mimura:** Data curation. **Hideyoshi Matsumura:** Supervision. **Hiroya Mizusawa:** Supervision; writing – review and editing.

## FUNDING INFORMATION

No source of funding.

## CONFLICT OF INTEREST STATEMENT

There is no conflict of interest to be declared.

## CONSENT

Written informed consent was from the patient's parent to publish this case report in accordance with the journal's patient consent policy.

## Data Availability

The data that support the findings of this study are available from the corresponding author upon reasonable request.
